# The microenvironment in the development of MASLD-MASH-HCC and associated therapeutic in MASH-HCC

**DOI:** 10.3389/fimmu.2025.1569915

**Published:** 2025-04-30

**Authors:** Qiulin Wu, Yan Yang, Shixun Lin, David A. Geller, Yihe Yan

**Affiliations:** ^1^ Department of General Surgery, The Second Affiliated Hospital of Guangxi Medical University, Nanning, Guangxi, China; ^2^ Thomas E. Starzl Transplantation Institute, Department of Surgery, University of Pittsburgh Medical Center, Pittsburgh, PA, United States

**Keywords:** microenvironment, MASLD, MASH, HCC, therapeutics

## Abstract

Metabolic dysfunction-associated steatotic liver disease (MASLD) is a series of obesity-related metabolic liver diseases, ranging from relatively benign hepatic steatosis to metabolic-associated steatohepatitis (MASH). With the changes in lifestyle, its incidence and prevalence have risen to epidemic proportions globally. In recent years, an increasing amount of evidence has indicated that the hepatic microenvironment is involved in the pathophysiological processes of MASH-induced liver fibrosis and the formation of hepatocellular carcinoma (HCC). The hepatic microenvironment is composed of various parenchymal and non-parenchymal cells, which communicate with each other through various factors. In this review, we focus on the changes in hepatocytes, cholangiocytes, liver sinusoidal endothelial cells (LSECs), hepatic stellate cells (HSCs), Kupffer cells (KC), dendritic cells (DC), neutrophils, monocytes, T and B lymphocytes, natural killer cells (NK), natural killer T cells (NKT), mucosal-associated invariant T cells (MAIT), γδT cells, and gut microbiota during the progression of MASLD. Furthermore, we discuss promising therapeutic strategies targeting the microenvironment of MASLD-MASH-HCC.

## Introduction

**Figure 1:**
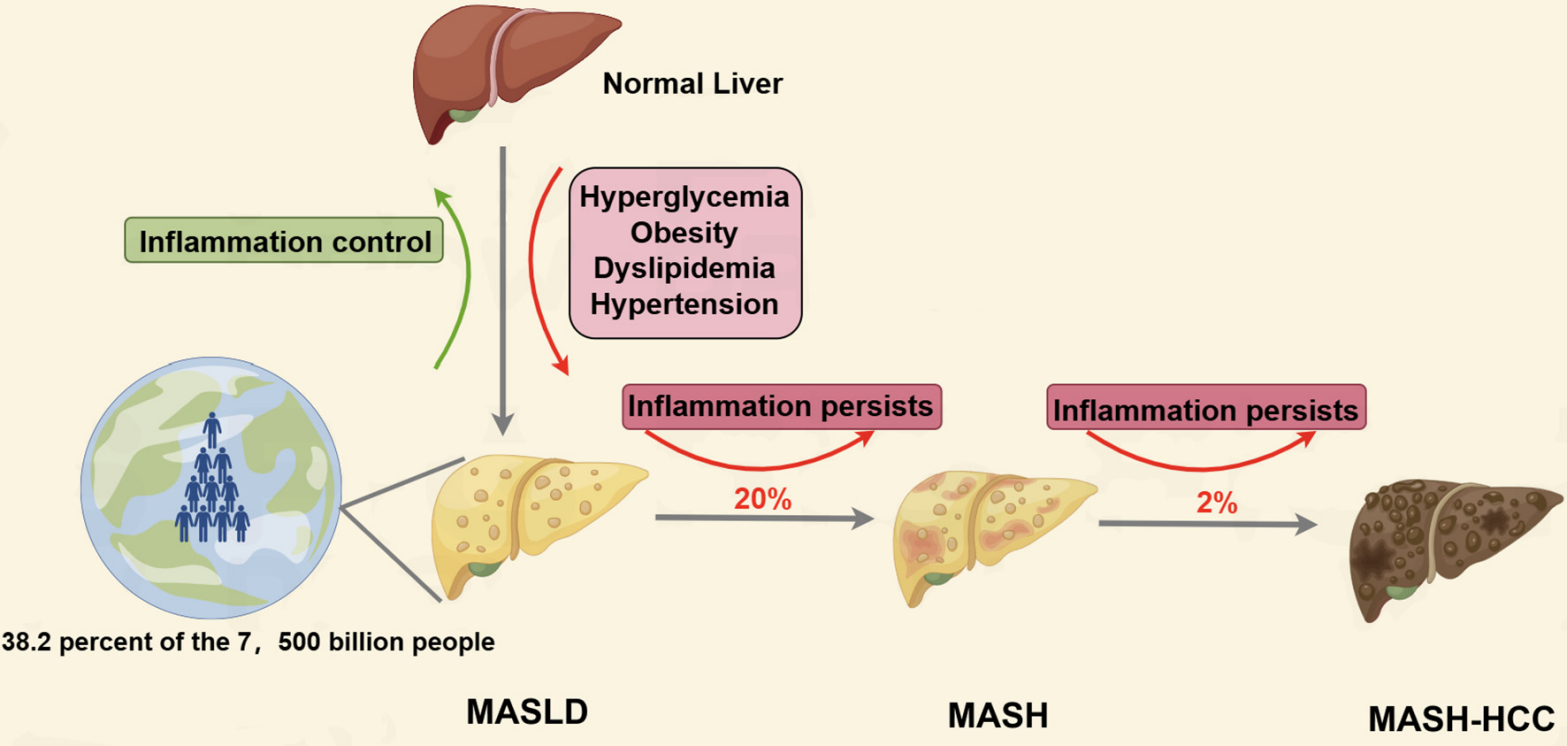
The development of MASLD, MASH and HCC.

Current findings reveal a global prevalence of metabolic dysfunction-associated steatotic liver disease (MASLD) at around 25% (1), with the Middle East exhibiting the highest prevalence at 32%, followed by South America at 31%, and Africa at the lowest at 14% (2). Over the past three decades, there has been a significant increase in MASLD prevalence, rising from 25.3% in 1990–2006 to 38.2% in 2016-2019 (3). Metabolic disorders such as hyperglycemia, obesity, dyslipidemia, and hypertension are known contributors to the development of MASLD (4). MASLD is characterized by hepatic steatosis, defined as >5% accumulation of triglycerides in hepatocytes. In contrast, metabolically associated steatohepatitis (MASH) is identified by >5% hepatic steatosis accompanied by hepatocytes damage, inflammation, and potentially fibrosis (5). Approximately 20% of MASLD cases progress to MASH, with a subset of MASH patients (about 2%) advancing to MASH-associated hepatocellular carcinoma (MASH-HCC) (see **Figure 1** (5). Following the rebranding of the American Association for the Study of Liver Diseases (AASLD) from non-alcoholic fatty liver disease (NAFLD) to MASLD and from non-alcoholic steatohepatitis (NASH) to MASH, the new nomenclature has garnered significant recognition among scholars for its enhanced applicability in both clinical and academic settings.

Approximately 10% (range 1-38%) of liver cancer cases are linked to MASLD, with higher prevalence rates (>20%) observed in studies conducted in the US, UK, India, Germany, and the Middle East, and lower rates (1-2%) reported in China and Japan (6). Utilizing mathematical models to forecast the incidence of MASH-HCC, projections indicate a substantial increase between 2016 and 2030, with a predicted rise of 47% in Japan, 82% in China, 88% in the UK, 117% in France, and 130% in the USA (7). Despite MASH-HCC having a lower incidence compared to virus-induced hepatocellular carcinoma (virus-HCC), the escalating prevalence of MASLD in conjunction with advancements in viral hepatitis treatment is anticipated to elevate its relative proportion and incidence (5).

**Figure 2:**
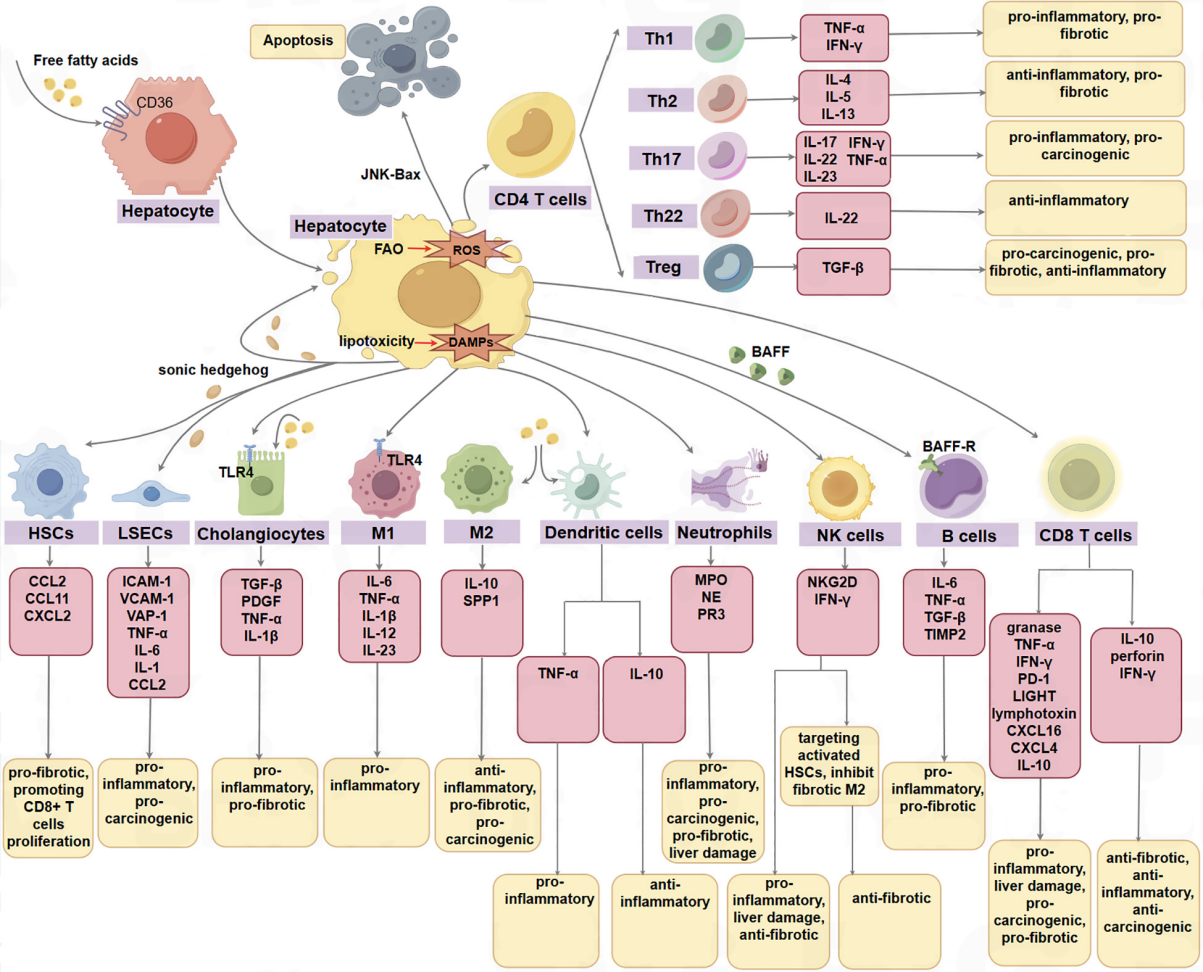
The parenchymal cells, non-parenchymal cells, and the extracellular matrix in the hepatic microenvironment function in the progression of MASLD, MASH, and MASH-HCC. Some cells have opposite effects in different phenotypes or disease stages, such as cx3cr1-expressing myeloid DCs can produce TNF-α to promote inflammation, while CD103+ dc plays a protective role in steatohepatitis by producing IL10. In addition, CD8T cells can not only promote the progression of NALFD by producing a series of inflammatory factors such as granase, TNF-α, IFN-γ, but also play an anti-inflammatory and anti-tumor role by producing IL-10 and perforin. M1 M1 macrophage, M2 M2 macrophage.

Novel insights propose that the liver microenvironment could represent a pivotal determinant in the evolution of MASLD, MASH, and MASH-related HCC, comprising parenchymal cells, non-parenchymal cells, and the extracellular matrix (see **Figure 2**). Parenchymal cells, such as hepatocytes and cholangiocytes, are integral components of the liver microenvironment. Non-parenchymal cells, including liver sinusoidal endothelial cells (LSECs), hepatic satellite cells (HSCs), Kupffer cells (KCs), dendritic cells (DCs), neutrophils, monocytes, T and B lymphocytes, natural killer cells (NK), natural killer T cells (NKT), mucosal-associated invariant T cells (MAITs), and γδT cells, are believed to have significant implications in the initiation and progression of HCC (8).

The current therapeutic strategies for MASH-HCC mirror those employed for other etiologies of HCC, encompassing transplantation, resection, or locoregional therapies for early or intermediate-stage disease (9). However, due to the distinct pathological characteristics of MASH-HCC, its tumor microenvironment differs substantially from virus-induced HCC. Consequently, while the prognosis following local treatment for early or intermediate-stage MASH-HCC and virus-induced HCC is generally comparable, advanced MASH-HCC exhibits limited efficacy and survival rates in immune checkpoint blockade (ICB) therapy (10, 11). Therefore, further investigations into the intricate interplay of the liver microenvironment in the pathogenesis of MASLD, MASH, and MASH-HCC are imperative for advancing our understanding of these conditions.

## Hepatocytes

The accumulation of lipids within hepatocytes is associated with lipotoxicity, leading to cellular stress and eventual cell death. This process triggers the release of damage-associated molecular patterns (DAMPs), which encompass a variety of molecules such as metabolites, microRNAs, mitochondrial double-stranded RNA, mitochondrial DNA, purine nucleotides, cytoplasmic proteins, and mitochondrial compounds (12). Metabolites considered as DAMPs mainly include fatty acid, uric acid, ceramide, cardiolipin, cholesterol, citrate, succinate, ATP, etc. All of these can induce the activation of the NLRP3 inflammasome (13). Subsequently, these DAMPs are recognized by members of the Toll-like receptor (TLR) family, such as TLR2, TLR4, TLR5, and TLR9 (14), leading to the activation of Kupffer cells and the recruitment of monocyte-derived macrophages (MDMs) and other immune cells to orchestrate a sterile inflammatory response aimed at restoring tissue homeostasis. For example, DAMPs can recruit KCs through TLR on the cell surface and activate them to release tumor necrosis factor-α (TNF-α), IL-1β, CCL2, etc. DAMPs can also recruit mast cells through TLR on the cell surface and activate them to release TNF-α, IL-1β, TGFβ, etc. Additionally, DAMPs can recruit DCs through TLR on the cell surface and activate them to release TNF-α and IL-1s (14). However, in cases where the proinflammatory stimulus persists, the inflammatory response may become chronic, leading to tissue remodeling, fibrosis, and sustained inflammation.

Furthermore, hepatocytes apoptosis is considered a pivotal mechanism contributing to liver fibrosis in the pathogenesis of MASH. Macrophages and apoptotic hepatocytes are significant sources of reactive oxygen species (ROS) production (15). The entry of long-chain fatty acids (LCFAs) into hepatocytes mitochondria via FAT/CD36 facilitates fatty acid oxidation (FAO), consequently increasing ROS generation (16). Elevated levels of ROS have the potential to activate c-Jun N-terminal kinase (JNK), also known as stress-activated protein kinase. The activation of JNK by ROS promotes the lipoapoptosis of hepatocytes by upregulating the pro-apoptotic Bcl-2 protein Bax, thereby initiating the mitochondrial apoptosis pathway (17). Conversely, ROS can also stimulate AMP-activated protein kinase (AMPK), which acts to suppress ROS production, thereby preserving metabolic equilibrium and cell survival. Notably, AMPK activity is diminished in MASLD and MASH, with AMPK demonstrating the ability to alleviate liver fat accumulation and MASH-associated hepatocytes apoptosis (18).

Ballooned hepatocytes represent a characteristic feature of MASH and are implicated in the pathogenesis of the disease. Hedgehog (Hh), a ligand involved in the developmental hedgehog signaling pathway (19), is released from distended hepatocytes, leading to the activation of LSECs and HSCs (20). However, dysregulated activation of the Hedgehog signaling pathway during MASH and fibrogenesis may ultimately contribute to the development of liver cancer (21).

## Cholangiocytes

Elevated levels of fatty acids (FAs) have been demonstrated to induce cholangiocytes lipoapoptosis in a FoxO3/miR-34a-dependent manner. MASLD patients present heightened levels of Ductular reaction (DR) and fibrosis, serving as markers of cholangiocytes damage. DR and biliary senescence are recognized as characteristic features of cholangiopathies, with their prevalence heightened in individuals with MASLD and MASH, suggesting a pivotal role in the progression of MASLD (22).

During the development and progression of MASLD, hepatocyte-derived DAMPs have been observed to interact with TLR4 expressed by biliary epithelial cells, leading to the manifestation of duct reactions and biliary senescence (23). Senescent cholangiocytes exhibit an augmented secretion of senescence-associated secretory phenotype (SASP) factors, including transforming growth factor-beta (TGF-β), platelet-derived growth factor (PDGF), TNF-α, and IL-1β (24). This increased secretion plays a role in the attraction and infiltration of immune cells, thereby worsening microvesicular steatosis and fibrosis as MASLD progresses.

NKT cells have been shown to become activated upon recognition of lipid antigens through cluster differentiation 1 molecules on bile duct cells, leading to the release of inflammatory cytokines and active participation in the inflammatory response (25). Furthermore, both secretin (SCT) and its receptor (SCTR) exhibit upregulation in MASLD/MASH patients compared to healthy controls. Notably, mice lacking SCT or SCTR demonstrated reduced levels of DR and biliary cell senescence, concomitant with an upregulation of miR-125b, a specific inhibitor targeting various enzymes involved in lipid biosynthesis, such as Elovl1 (26).

## LSECs

Under normal physiological circumstances, LSECs orchestrate a highly immunosuppressive microenvironment that acts as a protective shield against intestinal antigen-induced inflammation (27). In the initial phases of MASLD, LSECs demonstrate a downregulation of pro-inflammatory chemokines via a mitogen-activated protein kinase (MAPK)-dependent pathway, including CCL2, potentially serving as a compensatory mechanism to impede disease progression (28). The above conclusion is contrary to that CCL2 is upregulated in other cells in MASLD. The downregulation of CCL2 in LSECs may be related to the enhanced autophagy of LSECs in the early stage of MASLD, which aims to maintain cellular homeostasis (29). However, as MASH advances, LSECs transition to a pro-inflammatory state characterized by the heightened expression of adhesion molecules and pro-inflammatory mediators, such as intercellular adhesion molecule-1 (ICAM-1), vascular cell adhesion molecule-1 (VCAM-1), vascular adhesion protein-1 (VAP-1), TNF-α, IL-6, IL-1, and CCL2 (30, 31). Consequently, dysfunctional LSECs contribute to the inflammatory cascade and activation of KCs (32), thereby accelerating the progression of MASLD towards MASH and HCC (33). Furthermore, lipotoxicity has been shown to induce the generation of ROS by LSECs, thereby fostering a pro-carcinogenic milieu (31).

In addition to alterations in phenotype and functionality, the morphology of LSECs undergoes changes during the progression from MASLD to MASH. A prominent morphological change observed in LSECs is capillarization, which manifests early in the development of MASLD (34). The capillarization of LSECs hampers the exchange of substances between sinusoids and blood, obstructs the removal of hepatocyte-derived very low-density lipoprotein (VLDL), and leads to the accumulation of cholesterol and triglycerides within the liver (35), exacerbating hepatocellular steatosis (36). Moreover, capillarization reduces the transfer of chylomicrons to hepatocytes, stimulates new adipogenesis, and triggers a compensatory rise in cholesterol and triglyceride synthesis in hepatocytes (37). Notably, primary human LSECs exposed to oxidized low-density lipoprotein (LDL) exhibited reduced fenestrae diameter and porosity (38), while a 48-hour fasting period in rats led to an enlargement in the diameter of LSEC fenestrae (39). Additionally, consumption of a high-fat diet has been linked to a reduction in both the size and number of fenestrae in LSECs (40).

## HSCs

The potential dual role of HSCs in MASH has been postulated, suggesting a beneficial impact in the early stages and a deleterious effect in the later stages of the disease progression (41). In the context of MASLD, HSC activation is triggered by hepatocellular-derived Hh ligands and osteopontin (OPN), leading to the upregulation of transcription factors ETS1 and RUNX1 in HSCs (42, 43). Utilizing single-cell RNA sequencing (scRNA-seq) analysis in murine MASH models, it was observed that activated HSCs play a role in modulating macrophage functions through the secretion of stellakines, including CCL2, CCL11, and CXCL2 (44). Furthermore, HSC activation is induced by the secretion of IL-10 from CD8+ T cells, subsequently promoting CD8+ T cells proliferation and exacerbating MASH progression (45). HSCs are pivotal in the development of liver fibrosis (46), with quiescent HSCs capable of differentiating into myofibroblasts in response to hepatocyte-derived lipid mediators and pro-fibrotic cytokines such as TGF-β in MASH (47). The therapeutic targeting of both apoptosis and senescence of activated HSCs represents crucial strategies in mitigating hepatic fibrosis (47).

However, significant upregulation of key signaling molecules, including SHh, β-catenin, and Wnt10b, involved in the Hedgehog and Wnt signaling pathways, was observed in senescent HSCs. These findings suggest a potential role for these molecules in the process of hepatocellular malignant transformation within a microenvironment characterized by steatosis, inflammation, and fibrosis (48).

## Gut microbiota

The composition of gut microbiota has been shown to influence the immunological landscape of the liver, impacting both adaptive immunity (Tregs, Th1, Th2, Th17, NKT, and B cells) and innate immunity (Neutrophils, KCs, dendritic cells, and NK cells) (49). Dysbiosis of the gut microbiota is frequently observed in MASLD (50), with MASH patients exhibiting increased levels of *Actinomyces*, *Ruminococcus*, *Blautia*, and *Dorea*, and decreased levels of *Bacteroides* compared to healthy individuals (51). Conversely, alternative studies have reported lower levels of *Faecalibacterium* and *Anaerosporobacter* but higher levels of *Parabacteroides* and *Allisonella* in MASH patients (52). Furthermore, MASH fibrosis has been associated with higher levels of *Ruminococcus*, *Proteobacteria*, and *Escherichia coli*, and lower levels of *Firmicutes* (53).

In addition, the diameter and quantity of LSECs may exhibit a negative correlation with the presence of *Bacteroides* and a positive correlation with the abundance of *Firmicutes* (40). Furthermore, an elevated prevalence of alcohol-producing bacteria has been noted in individuals with MASH, suggesting a potential involvement of these bacterial strains in the pathogenesis of MASH (54). Recent research involving animal models has demonstrated that *Faecalibacterium prausnitzii* can mitigate inflammation in adipose tissue and enhance liver function in MASH mice (55).

The migration of bacterial byproducts from the gut microbiota, such as lipopolysaccharide (LPS), peptidoglycan, and bacterial DNA, through the portal vein can activate TLRs on KCs, triggering an inflammatory response and exacerbating MASH (56). Notably, individuals with MASLD often experience bacterial overgrowth, leading to heightened levels of bacterial products like LPS (57). Activation of KCs occurs upon binding of LPS to TLR4 (58). Oral administration of *Lactobacillus casei* to mice has been shown to suppress methionine-choline-deficient (MCD) diet-induced MASH by inhibiting TLR-4 signaling, resulting in reduced serum LPS levels, decreased liver inflammation, and fibrosis (59).

Recent findings suggest that the gut microbiota may play a role in the development of MASH-HCC by virtue of the immunomodulatory properties of their metabolites (50). Studies have linked HCC with alterations in gut microbiota composition and inflammation in MASLD patients, characterized by decreased levels of *Bifidobacterium* but increased abundance of *Bacteroides* and *Ruminococcaceae* (60). Furthermore, a recent investigation revealed an enrichment of short-chain fatty acid (SCFA)-producing bacteria in patients with MASLD-HCC, leading to an immunomodulatory shift towards immunosuppression marked by elevated IL-10+ Tregs and reduced CD8+ T cells (61). Notably, interventions such as antibiotic administration and intestinal sterilization have been shown to decrease the incidence of HCC in obese mice, underscoring the significant role of microbiota dysbiosis in the pathogenesis of HCC (62).

## Macrophages

In the conventional understanding, liver macrophages are primarily composed of liver-resident KCs and circulating MDMs (63). In patients with MASLD, the infiltration of portal macrophages is detected at an early stage, preceding the onset of inflammation, and their activation plays a role in the initiation and advancement of the disease (64). The primary mechanism underlying this process appears to involve lipid accumulation leading to the depletion of KCs in the liver. In MASH, hepatocytes damage induces the recruitment of CCR2+/CCR3+ MDMs by motivating KCs to release proinflammatory chemokines such as CCL2, CCL5, and CXCL10. As MASH progresses, KCs are gradually replaced by Ly-6C+ monocytes (65). These MDMs demonstrate reduced efficacy in promoting liver triglyceride storage and exacerbate liver damage in the context of MASH. This phenomenon is supported by the significant decrease in liver inflammation observed in CCR2-/- and CXCR3-/- MASH mice (66). Apart from MDMs, KCs have the capacity to recruit other cell types that contribute to the acceleration of MASH progression (67).

Furthermore, liver macrophages have the capacity to secrete a diverse array of cytokines that play a role in driving the progression of MASH. The balance between M1 and M2 phenotypes within hepatic macrophages is disrupted by hepatic steatosis, influenced by factors such as cytokines, lipid metabolites, and various signaling molecules (68). In the context of MASLD, KCs are activated upon recognition of DAMPs via TLR4 receptors (69). Upon activation, KCs release cytokines such as IL-6, TNF-α, and IL-1β, which in turn enhance the infiltration of NKT and CD8+ T cells into the liver, thereby promoting the progression of MASH (70).

Additionally, the unsaturated fatty acid oleic acid (OA) has been found to induce M2 macrophage polarization in the context of MASLD -MASH (71). The polarization of macrophages towards the M2 phenotype has been implicated in the promotion of MASH-associated liver cancer by creating an immunosuppressive microenvironment (72). Consequently, it has been postulated that the activity of HSCs may exhibit a beneficial effect in the early stages of MASH but a detrimental effect in the later stages (63). This suggests a potential transition from a pro-inflammatory M1 macrophage phenotype to an anti-inflammatory M2 macrophage phenotype during the progression of MASLD-MASH, mainly attributed to the secretion of IL10 by M2 macrophage, which activates arginase in M1 macrophage and promotes M1 apoptosis (73).

In the context of MASH, M2-type KCs exhibit the ability to mitigate hepatic inflammation by suppressing the activation of M1-type KCs, yet they possess the potential to promote fibrosis (74). Various investigations have delineated a distinct subset of macrophages characterized by the expression of myeloid cells 2 (TREM2), CD9, GPNMB, and SPP1 within the liver affected by MASH (75). These TREM2-expressing macrophages demonstrate elevated levels of CCR2 and CX3CR1 and are recruited to hepatic crown-like structures (CLSs) in the steatotic liver in a CCR2-dependent manner (76). Correspondingly, a separate scRNA-seq analysis has demonstrated a pathogenic subset of TREM2+CD9+ macrophages within the fibrotic microenvironment of human livers affected by MASH, exhibiting a positive correlation with the degree of MASH-induced liver fibrosis (77). Conversely, studies have revealed that the absence of TREM2 accelerates the progression of MASLD in murine models, potentially due to its anti-inflammatory properties. Mechanistically, TREM2 interacts with and signals through DAP12, leading to the downregulation of inflammatory gene transcription, such as TNF-α, IL-1β, and NOS2 (78).

Consequently, investigations have demonstrated that heightened recruitment of TREM2+ macrophages within the hepatic milieu and elevated levels of soluble TREM2 in the systemic circulation are linked to favorable prognoses in individuals afflicted with fibrotic MASH (79). Furthermore, prolonged exposure to excessive nutrition in the context of fatty liver conditions results in the compromise of TREM2-dependent macrophage efferocytic function, consequently exacerbating hepatic inflammation and the advancement of MASH (80). Noteworthy is the observation that the uptake and accumulation of fatty acids prompt macrophage polarization towards an immunosuppressive phenotype in HCC (81).

## Dendritic cells

Alongside macrophages, the hepatic environment harbors a population of DCs comprising distinct subsets of classical and plasmacytoid DCs (pDCs) that are situated in the subcapsular region, interstitial spaces between hepatocytes, and within the vasculature (82). Investigations have revealed that DCs with lower lipid content exhibit a more tolerogenic phenotype, capable of promoting Tregs induction, whereas lipid-rich hepatic DCs possess immunogenic properties, stimulating the activation of T cells, NK cells, and NKT cells (83). Recent findings have indicated an increase in CX3CR1-expressing myeloid DCs in mice subjected to a MCD diet, which led to heightened TNF-α production; conversely, blockade of CX3CR1 resulted in amelioration of steatohepatitis in mice, implicating a disease-promoting role for myeloid DCs in MASLD (84). Utilizing scRNA-seq and transcriptional network analyses, it was demonstrated that conventional type 1 dendritic cells (cDC1s) are more prevalent and exhibit enhanced production of proinflammatory cytokines and chemokines in both human and murine MASH (85). These XCR1+ cDC1s were found to drive inflammatory T cell activation and exacerbate MASH liver pathologies in murine models (86).

Conversely, a reduction in the frequency of pDCs is observed in MASH patients, with the decline in pDCs correlating inversely with the extent of liver damage as indicated by serum alanine transferase levels (87). A study utilizing the MCD diet reveals an increase in DCs within the liver of mice; however, following diphtheria toxin-induced depletion of CD11c+ cells, the severity of steatohepatitis worsens, and DC ablation leads to the proliferation of CD8+ T cells and reduction in Tregs, indicating a potential regulatory function (88). Conversely, during the transition from MASH to HCC, hepatic lipid accumulation may exacerbate DC impairment by enhancing triglyceride uptake via scavenger receptor A, thereby compromising tumor-specific antigen presentation to CD8+ T cells and anti-tumor immune responses (89). A targeted investigation has demonstrated that mice lacking CD103+ DCs exhibited more pronounced steatohepatitis, underscoring a protective role for CD103+ conventional dendritic cells (cDCs) (86). The divergent outcomes regarding DC involvement in MASH could be attributed to variations in distinct DC subtypes.

## Neutrophils

In MASH, there is an upregulation of key chemokines that attract neutrophils from hepatocytes (90). The extent of neutrophil infiltration is positively associated with the severity of MASLD (64). The involvement of neutrophils in MASLD is widely supported by the observation of elevated levels of Myeloperoxidase (MPO) (91), neutrophil elastase (NE) (64), and proteinase 3 (PR3) (92), as well as neutrophil extracellular traps (NETs) (93) in MASLD patients, all of which have been implicated in promoting liver injury and the progression of MASH. Apart from their role in activating HSCs and driving liver fibrosis through the induction of M2-macrophage polarization (90), MPO has been implicated in the formation of CLS in MASH (94). NE has been shown to modulate insulin signaling, with its deletion leading to improved insulin sensitivity in an obesity mouse model (95). Modulating the expression or activity of MPO, NE, or PR3 through genetic manipulation or pharmacological approaches presents a promising therapeutic strategy for ameliorating pathological changes in MASH (64, 96). NETs represent a novel mechanism for neutrophil elimination, with elevated free fatty acids (FFAs) triggering NETs formation in neutrophils, and inhibition of NETs resulting in reduced severity of steatohepatitis (93). Indeed, NETs formation appears to foster an immune-suppressive milieu through programmed death ligand 1 (PD-L1) signaling, contributing to T-cell exhaustion in murine models of MASH-induced HCC. Specifically, NETs promote Treg differentiation by binding and activating TLR4 in immature CD4+T cells to enhance mitochondrial OXPHOS, while Treg can inhibit Teffs’ inhibitory effect on tumors (97). Consequently, beyond their traditional role in immune response, neutrophils are now recognized as potential mediators of MASH-HCC progression.

## NK cells

In individuals with MASH, elevated levels of hepatic CXCL9, CXCL10, CXCL11, CCL3, CCL4, and CCL5 were observed compared to healthy controls, with circulating CXCL10 levels correlating with the severity of lobular inflammation (66). Following MCD treatment, increased CXCL10 expression facilitated the recruitment of NK cells to the liver (98). The upregulation of chemokine expression, including CXCL10, CCL2, and CCL5, was found to be reliant on IL-15 signaling in hepatocytes in response to a high-fat diet (HFD), with IL-15-deficient mice exhibiting reduced MASH severity and diminished NK cell presence in the liver (99). Similarly, NK cells are activated by cytokines such as IL-12, IL-15, and IL-18 to produce interferon-gamma (IFN-γ), thereby driving the progression from MASLD to MASH (99).

Studies have revealed a notable increase in the population of NK cells expressing nature killer group 2D (NKG2D) in MASH compared to healthy individuals (100). Additionally, elevated levels of the NKG2D ligand major histocompatibility complex (MHC) class I associated chains A and B (MICA/B) in the liver have been observed in MASH patients, suggesting a potential involvement of NK cells in MASH progression through interactions with MICA/B (101). This interaction may trigger JAK-STAT1/3 and nuclear factor κB (NF-κB) signaling pathways in the liver, leading to hepatocytes damage (100). Interestingly, our investigations have demonstrated that NK cells exhibit cytotoxic activity against MICA-expressing cancer cells via the NKG2D receptor during HCC progression (102). Conversely, NK cells may play a protective role in the development of MASH-related fibrosis. Notably, NK cells have been shown to regulate liver fibrosis by directly targeting activated hepatic stellate cells through receptors such as NKG2D and NKp46, as well as the p38/PI3K/AKT pathway (103).

A recent investigation has reported a negative correlation between liver stiffness measurement and the total number of NK cells (104). In a murine model utilizing the MCD diet, an increase in CD49b+ NKp46+ conventional NK (cNK) cells is observed to inhibit fibrotic M2 phenotypic differentiation by skewing macrophages towards M1-like phenotypes. This effect is dependent on the cNK cells’ secretion of IFN-γ rather than granzyme-mediated cytotoxicity (105).

## B cells

B lymphocytes constitute approximately 6% of intrahepatic cells and represent around 50% of intrahepatic lymphocytes in mice (106). Focal clusters of B cells have been identified in the livers of both MASH patients and mice (107), which secrete pro-inflammatory cytokines such as TNF-α and IL-6 (108). While it is commonly believed that B cells produce fewer inflammatory cytokines compared to macrophages and neutrophils, a subset of B cells expressing high levels of BAFF-R has been detected during MASH. Upregulated BAFF in the liver may serve as one of the cytokines that facilitate the maturation and activation of these B cells (109). Treatment with BAFF depletion or induction of B-cell deficiency has been shown to ameliorate steatohepatitis in mice (110).

In MASH, B cells exhibit not only the capacity to generate pro-inflammatory mediators but also to participate in antigen presentation. The activation of B cells during MASH is mechanistically linked to signaling pathways involving the innate adaptor protein myeloid differentiation primary response protein 88 (MyD88). Specifically, the deletion of MyD88 in B cells has been shown to diminish hepatic T cell-mediated inflammation and fibrosis (109). Furthermore, RNA-sequencing analyses have unveiled that intrahepatic B cells play a role in MASH by expressing genes associated with fibrosis, such as TGF-β and TIMP2 (111). The pro-fibrotic actions of B cells are mediated through the production of pro-inflammatory molecules that stimulate HSCs and macrophages. Activated HSCs, in turn, promote the survival and maturation of liver B cells by releasing retinoic acid. Notably, the presence of circulating IgA has been identified as an independent predictor of advanced fibrosis and HCC development in MASH (111). Collectively, these findings underscore the dual role of B cells in driving inflammation and fibrosis progression in MASH.

## CD8+ T cells

The presence of ROS and its byproduct derived from lipid peroxidation, malondialdehyde (MDA), has been shown to trigger the recruitment of CD8+ T cells to the liver in both human subjects and murine models of MASLD (10, 11, 67, 112). In response to metabolic triggers like acetate and extracellular ATP, CXCR6+CD8+ T cells demonstrate self-destructive tendencies regardless of MHC-class-I molecules. This behavior is characterized by increased expression of granase, TNF-α, IFN-γ, and programmed death 1 (PD-1), which is facilitated by IL-15-induced downregulation of the transcription factor FOXO1. Consequently, this cascade of events promotes hepatocytes injury and contributes to the development of MASH and HCC (10).

Furthermore, a study has demonstrated that the administration of anti-PD-1 immunotherapy to mice fed a choline-deficient high-fat diet (CD-HFD) led to an increase in hepatic resident-like (CXCR6+) and effector (GZMK+ and GZMA+) CD8+ T cells expressing PD-1. These cells are found to secrete tumor necrosis factor superfamily member 14 (TNFSF14/LIGHT) and lymphotoxin, which respectively activate the lymphotoxin β receptor (LTβR) and NF-κB signaling pathways in hepatocytes. This activation serves as a crucial intrinsic regulatory mechanism in hepatocytes, influencing the transition from MASH to HCC (67). Moreover, B2 microglobulin knockout (b2m-/-) mice, which exhibit a severe deficiency in CD8+ T cells, are found to be protected from liver damage and tumorigenesis induced by a CD-HFD diet (67).

Additionally, in the murine model of MASH with cirrhosis, hepatic fibrosis has been linked to an elevated CD8+/CD4+ T cell ratio (113). Within the MASH mouse model, hepatic CD8+ T cells expressing CXCR1 and CXCR6 have been identified to effectively activate HSCs through the secretion of IL-10, CXCL4, and CXCL16. The reduction of CD8+ T cells within the MASH liver has been associated with decreased mRNA levels of fibrotic genes, including α-smooth muscle actin, TGF-β, collagen type 1 alpha 1, and collagen type 1 alpha 2 (112).

While the current evidence supports the pathogenic role of total CD8+ T cells in MASH progression, the application of single-cell genomics has unveiled functionally distinct subsets within the CD8+ T cell population. Through single-cell RNA analysis, researchers have characterized a subset of hepatic resident memory T cells marked by the expression of CD44+CD62L− CD69+CD8+ T cells, which also exhibit CXCR6. These resident T cells have been found to play a crucial role in resolving the fibrotic process. Specifically, these cells interact with HSCs through CCR5-driven chemotaxis and induce apoptosis in HSCs via Fas-FasL contact (114).

Concurrently, enhanced intrahepatic retention of activated NKT cells and CD8+ T cells has been observed to mitigate glucose intolerance, liver steatosis, the release of inflammatory factors (such as IL-10), and tumor growth (via the IRF1/CXCL10/CXCR3 axis) in MASH, consequently suppressing the development of HCC (115–117). Furthermore, CD8+ T cells have been identified to secrete perforin, which triggers apoptosis in M1 macrophages, thereby inhibiting the synthesis of pro-inflammatory cytokines in MCD-fed mice (118). Hence, it is evident that specific subsets of CD8+ T cells also exhibit a protective role in the context of MASH.

## CD4+ T cells

Linoleic acid has been shown to disrupt mitochondrial function in CD4+ T cells, culminating in elevated ROS generation, activation of caspases, and subsequent cell death, leading to an overall reduction in CD4+ T cell numbers (119). Despite the decline in the total count of CD4+ T cells observed in MASH and MASH-HCC, a specific subset of T helper (Th) cells has been found to increase (120). Based on their functional characteristics and cytokine secretion profiles, CD4+ T cells are further categorized into distinct subtypes including Th1, Th2, Th17, Th22, Tregs, and other subsets (121).

## Th1 cells

In MASLD, Th1 cells differentiate from naïve CD4+ T cells and secrete cytokines such as IFN-γ and TNF-α, exerting pro-inflammatory effects (122). Clinical evidence further substantiates these findings, demonstrating an increased proportion of liver and circulating Th1 cells producing IFN-γ in both pediatric and adult patients with MASH (123, 124). Additionally, elevated plasma levels of IFN-γ have been positively correlated with the abundance and size of hepatic lymphocyte clusters, as well as the severity of fibrosis in MASH (107). IFN-γ can activate KCs, leading to the progression of MASH and MASH-associated HCC (125). Furthermore, the IFN-γ-induced chemokine CXCL10 is capable of attracting T cells expressing the CXCR3 receptor, and the absence of CXCR3 or deletion of CXCL10 has been shown to alleviate liver inflammation, injury, and fibrosis (66). Studies have revealed an upregulation of the TNF family co-stimulatory molecule OX40 in CD4+ T cells of mice fed a high-fat diet, with OX40 deficiency resulting in reduced infiltration of CD4+ T cells and diminished Th1 cell differentiation, thereby ameliorating steatohepatitis (126).

## Th2 cells

Currently, limited research exists on the role of Th2 cells in MASLD and MASH, which predominantly secrete IL-4, IL-5, and IL-13 and activate signal transducer and activator of transcription (STAT) proteins 5 and 6 (124). While investigations have indicated an elevated Th1/Th2 ratio in mesenteric lymph nodes of MASLD-afflicted mice (127), a contrasting scenario is observed in peripheral blood of MASLD patients, where Th2 cell levels are heightened compared to healthy individuals (128). Notable differences in Th2 cell populations in peripheral blood or liver have not been observed among patients with MASH, MASLD, or healthy controls (124). Despite the potential anti-inflammatory role of Th2 cells in MASLD (129), their involvement in liver fibrosis progression, particularly in the presence of IL-13, is evident. Elevated levels of circulating IL-13 and increased expression of IL-13 receptor alpha 2 (IL-13Rα2) in the liver have been reported in individuals with MASH, with liver fibrosis mitigation observed upon targeting IL-13Rα2+ cells, including HSCs (130).

## Th17 cells

IL-17 secreted by Th17 cells exacerbates inflammation in MASLD by inducing ROS and enhancing neutrophil infiltration, thereby hastening disease progression (131). Both in MASLD patients and murine models, hepatic Th17 cell populations exhibit a continuous increase throughout disease advancement, peaking at the onset of steatohepatitis and later stages of the condition (132, 133). Steatotic hepatocytes display heightened sensitivity to IL-17A, leading to upregulation of its receptor IL-17RA, augmented cytokine release encompassing IL-6, TNF-α, CXCL1, and increased lipid synthesis (134). Recent investigations have unveiled that MASLD triggers a rise in circulating and liver-resident CXCR3+ Th17 cells through glycolysis induction, enhancing their capacity to produce IL-17A, IFN-γ, and TNF-α (134). Mice subjected to glycolytic inhibition or lacking IL-17A or its receptor, IL-17RA, exhibited milder steatohepatitis symptoms (131). Furthermore, Th17 cells prompt macrophages to release pro-inflammatory cytokines such as IL-6, TGF-β, TNF-α, and IFN-γ through IL-17, thereby amplifying the inflammatory cascade (135).

Studies have documented that the expansion of Th17 cells, along with their secretion of IL-17, IL-22, and IL-23, contributes to the promotion of hepatocarcinogenesis in MASH (136). In mice fed a CD-HFD diet, IL-17A released by hepatic Th17 cells induces insulin resistance in the adjacent adipose tissue, leading to enhanced influx of fatty acids into the liver, thereby triggering MASH-related HCC. Inhibition of IL-17A signaling mitigates liver damage and prevents the development of HCC (137).

## Th22 cells

Within the context of MASH, CD4+ T cells producing IL-22 (Th22) are notably abundant during the initial and subsequent expansions of Th17 cells (129). IL-22 has been implicated in exerting inhibitory and protective effects on the advancement of MASLD. Administration of recombinant IL-22 has shown significant improvement in the liver damage and steatohepatitis in animal models of MASH, potentially mediated by STAT3 pathway (138). IL-22 also demonstrates the ability to mitigate lipotoxicity induced by palmitate by inhibiting JNK in a phosphoinositide 3-kinase (PI3K)/protein kinase B (AKT)-dependent manner (129). Notably, the beneficial effects of IL-22 are observed in the absence of IL-17, as IL-17 can upregulate phosphatase and tensin homolog (PTEN), an antagonist of PI3K-AKT signaling (129). Furthermore, IL-22 can upregulate the expression of anti-apoptotic genes such as bcl2 and suppress the expression of lipid biosynthesis genes such as scd1 (139).

## Tregs

In the MASH context, the generation of NETs has been demonstrated to promote the differentiation of Tregs from naive CD4+ T cells (93). Notably, the population of liver-resident Tregs (CD4+Foxp3+) expands during MASH, with FOXP3+ Tregs demonstrating the ability to suppress the proliferation and activity of CD8+ T cells and Th1 cells, which play a crucial role in immune surveillance against cancer. Notably, the depletion of Tregs (CD4+Foxp3+) has been found to significantly impede the progression of HCC in a MASH model induced by choline deficiency, high-fat diet consumption, and diethylnitrosamine administration (97). Conversely, the transfer of Tregs in MASH exacerbates the severity of the disease (140). Furthermore, Tregs are known to secrete TGF-β, which possesses pro-fibrotic properties (141). However, a decline in hepatic Treg numbers has been observed in MASLD, potentially attributed to the induction of Treg apoptosis by KCs and DCs through the generation of oxidative stress, TNF-α, and interferon I (142, 143).

Additionally, the upregulation of leptin production in obesity has been demonstrated to impede the differentiation of Tregs while concurrently promoting the activation of dendritic cells and the polarization of CD4+ T cells towards the Th1 and Th17 subsets (144). Remarkably, a recent investigation revealed that the reduction in Treg numbers in mice achieved through the combined deficiency of the co-stimulatory molecules CD80 and CD86 exacerbated adipose tissue inflammation and steatohepatitis induced by a high-fat diet (145). Moreover, the transfer of Tregs has been shown to mitigate TNF-α signaling induced by a high-fat diet and LPS-triggered hepatotoxicity (142). These seemingly contradictory findings may be ascribed to the dual role of Tregs in MASH, acting protectively during the inflammatory phase and promoting tumor development in later stages.

## Innate-like T lymphocytes

Hepatic innate-like T lymphocytes, such as γδT cells, NKT cells, and MAITs, have been implicated in the pathogenesis of MASH in humans and mice.

## γδ T cells

γδ T cells have been implicated in the advancement of MASLD. In murine models of MASH, there is a notable increase in the population of γδ T cells within the liver, contributing to the development of steatohepatitis, hepatic injury, and perturbed glucose metabolism via the secretion of IL-17A (146). The recruitment of γδ T cells, particularly IL-17high Ly6C− CD44+ γδ T cells, is dependent on the chemokine receptors CCR2 and CCR5 in the liver afflicted with steatohepatitis, facilitating the infiltration of inflammatory monocytes while dampening IFN-γ production in CD4+ T cells through the release of vascular endothelial growth factor (VEGF) and IL-15 (147). Conversely, the cytokines VEGF and IL-15 derived from γδ T cells in a CD1d-dependent manner have been shown to suppress IFN-γ, which exerts protective effects in certain cases of MASH (147). Additionally, γδ T cells can promote the progression of fibrosis by activating HSCs and KCs through the production of IL-17 (148). Notably, MASH mice lacking γδ T cells exhibits milder liver injury and steatohepatitis (149).

## NKT cells

An increase of 10% in NKT cells has been observed in the livers of individuals with cirrhosis unrelated to MASH, while a 20% increase is noted in those with MASH-related cirrhosis (150). In the context of MASH, NKT cells not only instigate inflammation and steatosis by releasing LIGHT, IFN-γ, TNF-α, and IL-17A upon recognition of lipid antigens but also motivate HSCs to promote fibrosis via the secretion of OPN and Hh ligands in response to IL-15 stimulation and Hh pathway activation (67, 150, 151). Mice lacking IL-15 or IL-15Rα exhibit reduced intrahepatic CD4+, CD8+, NKT cells, and HSCs and, when subjected to a high-fat diet, display less severe steatosis and lobular inflammation compared to their wild-type counterparts (152). While the aforementioned findings support the role of NKT cells in driving the progression of MASH, their impact on the development or inhibition of MASH-related liver cancer remains uncertain. Research has indicated that liver NKT cells secrete LIGHT, a ligand for the lymphotoxin beta receptor (LTβR), which activates NF-kB signaling by binding to LTβR on hepatocytes and collaborates with CD8+ T cells to facilitate malignant transformation (67). Notably, targeted deletion of LTβR on hepatocytes or depletion of NKT cells has been shown to mitigate high-fat diet-induced HCC (67).

The involvement of distinct subpopulations of NKT cells in the regulation of disease progression presents a complex scenario. Several investigations have documented a decrease in the abundance of NKT cells in both human and murine MASLD (11, 153). This reduction in NKT cell numbers has been linked to heightened apoptosis triggered by IL-12 produced by KCs (154) and intensified Tim-3/Gal-9 signaling pathways (155). Mice deficient in Ja18 and CD1d, which lack NKT cells, exhibit elevated rates of weight gain and liver steatosis in the context of MASH (10). Conversely, leptin-deficient mice demonstrate diminished hepatic steatosis following the transfer of NKT cells, potentially associated with the down-regulation of IL-10 (116). Moreover, the accumulation of hepatic cholesterol selectively impedes the anti-tumor immune surveillance function of NKT cells through the buildup of lipid peroxidation and SREBP2-dependent cytotoxic impairments in diet-induced mouse models of MASH-HCC (156).

## MAITs

CCR5 facilitates the migration of MAITs to sites of hepatic steatosis through its interaction with CCL5 (157). In individuals with MASLD-related cirrhosis, MALTs secrete IL-17 and TNF-α to stimulate the activation of hepatic fibrotic cells (158). Concurrently, the functions of circulating MALT cells undergo alterations, characterized by an increase in IL-4 secretion and a decrease in IFN-γ production (157). IL-4, known for its anti-inflammatory properties, may induce a shift KCs towards an M2 phenotype in the context of MASLD (73). This protective role is underscored by studies in animal models, where mice lacking MAITs exhibit pronounced hepatic steatosis (157) or exacerbated diabetes (159). In essence, MAITs appear to exert an anti-inflammatory protective function in MASLD while simultaneously promoting the progression of fibrosis.

## Therapeutic targeting the microenvironment of MASLD-MASH-HCC

For the treatment of MASH, the goal should be to improve clinical outcomes, that is, to reduce the MASH-related mortality rate and decrease the incidence of progression to cirrhosis and HCC. The only treatment that has been strongly proven to improve liver injury in patients with MASLD without severe hepatic fibrosis is weight loss through diet (160). In addition, some studies have shown that in some patients, after lifestyle intervention and Roux-en-Y gastric bypass, MASH has been improved. In these improved patients, the inflammatory-related pathways, such as the complement, TNF-α, and IL-6 signaling pathways, have all been inhibited, mainly manifested by the downregulation of CXCL9, CXCL10 and lysozyme (161).

With the in-depth research on the mechanisms of metabolic damage, as well as the activation of inflammatory and fibrotic pathways in MASH, some drugs targeting several cellular components or molecular pathways of MASH have been preliminarily explored. Resmetirom is a selective agonist of the thyroid hormone receptor β (THR-β) and is the first treatment approved by the US Food and Drug Administration (FDA) for MASH (162–164). In MASH, the function of THR-β in the liver is impaired, leading to reduced mitochondrial function and fatty acid β-oxidation, which in turn increases fibrosis. Stimulating THR-β can promote the regulation of hepatic lipid metabolism (165). THR increases cholesterol metabolism through the hepatic enzyme CYP7A1 and reduces *de novo* lipogenesis (165). In contrast, the thyroid hormone activity outside the liver, including in the heart and bones, is mainly mediated by thyroid hormone receptor α (THR-α) (166).

In a mouse model of MASH with fibrosis, Resmetirom treatment significantly improved the MASLD activity score and reduced hepatic fibrosis (167). Data from phase II/III clinical trials reflect the safety and potential efficacy of Resmetirom in adult patients with MASH (162, 163). The activation of THR-β in the liver by Resmetirom has been shown to reduce triglycerides by 30.8%, low-density lipoprotein cholesterol (LDLC) by 22.3%, and lipoprotein (a) by 37.9% (162). Both 80 mg and 100 mg of Resmetirom can not only significantly improve the progression of steatohepatitis (24.2% for 80 mg, 25.9% for 100 mg, compared with 14.2% in the placebo group), but also reverse the hepatic fibrosis in MASH (25.9% for 80 mg, 29.9% for 100 mg, compared with 9.7% in the placebo group) (168). Compared with the placebo, among every 1,000 patients treated with Resmetirom, the number of decompensated cirrhosis events decreases by 87, the number of HCC events decreases by 59, and the number of liver transplants decreases by 30 (169). However, for patients with decompensated cirrhosis (Child-Pugh class B or C), Resmetirom should be avoided because as the dosage of Resmetirom increases, the risk of adverse events also increases (170).

Since the immune response seems to play a key role in MASH and its progression to HCC, methods to address this issue need to be explored. Cenicriviroc, a CCR2/CCR5 antagonist targeting pro-inflammatory monocytes, has been shown in a mouse model. In a phase II clinical trial, it was confirmed that Cenicriviroc improved the fibrosis in patients with MASLD (171). However, in a larger-scale phase III (AURORA) clinical trial, after one year of treatment, Cenicriviroc did not show sustained antifibrotic efficacy, leading to the termination of the study on its monotherapy for MASH. In another phase II clinical trial, Cenicriviroc was combined with the farnesoid X receptor (FXR) agonist Tropifexor to improve the hepatic fibrosis in patients with MASH, but the result analysis is still ongoing (172). FXR is mainly expressed in the liver, intestine, kidney, adipose tissue, and immune cells, participates in lipid metabolism, and exerts anti-inflammatory and antifibrotic effects (173).

In another phase IIa clinical trial, patients with MASH and type 2 diabetes mellitus received the treatment of CD3 monoclonal antibody (OKT3) to induce Tregs, and the transaminase levels and insulin resistance were improved (174). Currently, a phase II clinical trial (NCT03291249) is ongoing to determine the safety and effectiveness of Foralumab, a new CD3 monoclonal antibody, in patients with MASH and type 2 diabetes mellitus (T2DM) (175). However, despite a large number of explorations in regulating the immune system to control the progression of MASLD, conclusive evidence is still lacking.

In the clinical trials of drug development for MASH, the improvement of hepatic fibrosis is often used as the primary or secondary endpoint. Given the central role of HSCs in hepatic fibrosis, inhibiting HSCs activation has long been proposed as a therapeutic strategy to prevent the progression of MASH-related fibrosis. In MASH, the activation of the Notch signaling pathway in hepatocytes induces an increase in the secretion of OPN, which in turn activates HSCs (42). Therefore, *Nicastrin* antisense oligonucleotide (*Ncst* ASO), an inhibitor of Notch, can reduce the fibrosis in MASH diet-fed mice by inhibiting the activation of HSCs through the secretion of OPN by hepatocytes (42).

In addition, in MASH, the nuclear factor of activated T cells 4 (NFATc4) in hepatocytes translocates from the cytoplasm to the nucleus, which can inhibit the transcriptional activity of peroxisome proliferator-activated receptor α (PPARα) and induce the expression of OPN. Inhibiting the activation of NFATc4 can alleviate the lipid deposition and the progression of fibrosis in MASH mice (176). Widjaja et al. developed a neutralizing anti-IL-11 antibody and a neutralizing anti-IL-11 receptor α (IL-11RA) antibody, and found that they significantly alleviated the hepatic steatosis, liver inflammation, and hepatic fibrosis in MASH mice by inhibiting the activation of HSCs by IL-11 (177). Protease-activated receptor-2 (PAR2) is an emerging new target expressed on hepatic stellate cells and hepatocytes, which can regulate liver injury, inflammation, and fibrosis (178). Pepducin PZ-235 is a complete antagonist of PAR2. Pharmacological inhibition of PAR2 can not only prevent the activation of HSCs and fibrosis, but also reduce the production of ROS mediated by hepatocytes through inhibiting PAR2 (178).

The therapeutic efficacy of MASH-related HCC is akin to virus-related HCC in surgical, liver transplantation, radiofrequency ablation (RFA), transarterial chemoembolization (TACE) interventions, and molecular targeted therapy (see **Table 1**) (179–185), but variations in the immune milieu result in divergent responses to ICB therapy. Although favorable outcomes are observed across different etiologies, a meta-analysis indicates that patients with MASLD etiology may derive comparatively lesser benefits from anti-PD-1 treatment. Notably, a combinatorial approach utilizing both anti-PD-L1 and anti-VEGF therapies has demonstrated enhanced survival rates (186).

**Table 1:** Therapeutic variance between MASH-HCC and virus-induced HCC.

Treatment	Date	n(MASLD/non- MASLD)	Outcome
Liver resection (179)	2022.11.29	11477(2470/9007)	Slightly higher overall survival (HR 0.87; 95% CI: 0.75–1.02) and recurrence-free survival (HR 0.93; 95% CI: 0.84–1.02) than those with HCC of other aetiologies.
All curative therapy (resection, transplantation, ablation) (180)	2021.01.13	5579(NA)	Liver resection: improved disease-free survival (HR 0.85; 95% CI: 0.74–0.98, p = 0.03) and overall survival (HR 0.87; 95% CI: 0.81–0.93; p < 0.0001) than those with HCC of other aetiologies. All curative therapy: improved overall survival (HR 0.96; 95% CI: 0.86–1.06; p = 0.40) and disease-free survival (HR 0.85; 95% CI: 0.74–0.98; p = 0.03) than those with HCC of other aetiologies.
Radiofrequency ablation (181)	2022.01.05	520(62/458)	There were no differences in morbidity, tumour recurrence and overall survival among patients with MASLD-HCC vs other aetiologies as well as no prognostic impact of metabolic components (p = 0.3168).
Transarterial chemoembolization (182)	2019.11.04	220(30/190)	There was a non-significant increased overall survival in the non-MASH [median 1078 days (95% CI: 668–1594)] as compared to the MASH cohort [median 706 days (95% CI: 314–not reached)] (p = 0.08)
ICIs, TKI and anti-VEGF (183)	2021.06.12	22113(NA)	ICIs: improved overall survival in patients with viral-related HCC (HR=0.64; 95% CI: 0.5–0.83) compared with nonviral-related HCC (HR=0.92, 95% CI 0.77–1.11) (p = 0.0259). TKI and anti-VEGF: no impact of etiology in outcome was observed with TKI/anti-VEGF therapies. The overall survival in patients with viral-related HCC (HR=0.81; 95% CI: 0.71–0.92) compared with nonviral-related HCC (HR=0.82; 95% CI: 0.67–1.01) (p = 0.8828).
Sorafenib (184)	2021.10.24	180(37/143)	There was no significant differences in overall survival between the Virus/alcohol group and the MASLD/MASH group in patients who received sequential therapy (median survival times was 23.4 and 27.0 months. p = 0.173, respectively).
Lenvatinib (185)	2021.11.27	1232(236/996)	MASH-HCC was associated with longer overall survival (22.2 versus 15.1 months; HR 0.69; 95% CI: 0.56-0.85; p = 0.0006). MASH-HCC was associated with longer disease-free survival (7.5 versus 6.5 months; HR 0.84; 95% CI: 0.71-0.99; p = 0.0436).

ICI, immune checkpoint inhibitor; TKI, tyrosine kinase inhibitor.

In a murine model of MASH-HCC, treatment with anti-PD-1 immunotherapy results in an escalation in the occurrence of MASH-HCC and the quantity and dimensions of tumor nodules. This augmentation is linked to heightened levels of hepatic CD8+ PD1+ T cells and TNF+ T cells, and is averted by the depletion of CD8+ T cells or TNF+ neutralization (10). The primary rationale behind this phenomenon may stem from the fact that liver tumors in mice treated with anti-PD1 exhibited an elevated presence of CD8+/PD1+ T cells, which, in contrast to resident hepatic CD8+ T cells, display amplified expression of effector and exhaustion markers alongside impaired proliferative capacity. These CD8+/PD1+ T cells not only exhibit compromised immune surveillance capabilities but also possess tissue-damaging effects (11). This mechanistic insight could elucidate the observed acceleration in tumor progression in around 13% of advanced HCC patients undergoing anti-PD-1 therapy, a phenomenon recognized as hyperprogressive syndrome (11). Consequently, the imperative for improved stratification of HCC patients undergoing immunotherapy based on the etiology of liver cancer is underscored, necessitating the development of tailored immunotherapeutic strategies for MASH-HCC.

## Conclusion

MASH represents a significant global health challenge and is anticipated to emerge as a prominent contributor to HCC incidence with the rise in metabolic disorders (5). The transition from MASH to HCC is governed by a multitude of factors encompassing molecular modifications, germline variability, fibrotic conditions, immune milieu, and the microbiome. All cellular constituents implicated in MASLD, MASH, and MASH-related HCC, such as T lymphocytes, macrophages, hepatocytes, and hepatic stellate cells, exhibit immunomodulatory characteristics. Numerous investigations have underscored the presence of a distinct immune cell subset in MASH-related HCC in contrast to virus-induced HCC, partially elucidating the dissimilarities in immune checkpoint inhibitor responsiveness between the two conditions. Nonetheless, the intricate interplay among tumor cells, immune cells, and stromal cells remains largely unexplored. Consequently, further exploration into the variances in the microenvironment between MASH-related HCC and virus-induced HCC is imperative to unravel the distinctions in treatment outcomes, facilitating the implementation of more efficacious, personalized therapeutic strategies in the foreseeable future.

## Glossary

AASLD: American Association for the Study of Liver Diseases
AMPK: AMP-activated protein kinase
ASH: Alcoholic steatohepatitis
cDC1s: Conventional type 1 dendritic cells
CD-HFD: Choline-deficient high-fat diet
CTLA-4: Cytotoxic T-lymphocyte-associated protein 4
CHB: Chronic hepatitis B
DCs: Dendritic cells
DAMPs: Damage-associated molecular patterns
DR: Ductular reaction
FABP4: Fatty acid-binding protein 4
FAO: Fatty acid oxidation
FDA: Food and Drug Administration
FFAs: Free fatty acids
HBV: Hepatitis B virus
HCV: Hepatitis C virus
HCC: Hepatocellular carcinoma
Hh: Hedgehog
HFD: High-fat diet
ICB: Immune checkpoint blockade
ICAM-1: Intercellular adhesion molecule-1
IFN-γ: interferon-gamma
IL-13Rα2: IL-13 receptor alpha 2
JNK: C-Jun N-terminal kinase
KCs: Kupffer cells
LSECs: Liver sinusoidal endothelial cells
LCFAs: Long-chain fatty acids
LDLC: low-density lipoprotein cholesterol
LDL: Low-density lipoprotein
LPS: Lipopolysaccharide
LTβR: Lymphotoxin β
MAPK: Mitogen-activated protein kinase
MASLD: Metabolic dysfunction-associated steatotic liver disease
MASH: Metabolically associated steatohepatitis
MASH-HCC: MASH-associated hepatocellular carcinoma
MAITs: Mucosal-associated invariant T cells
MDMs: Monocyte-derived macrophages
MCD: Methionine-choline-deficient
MPO: Myeloperoxidase
MICA/B: Major histocompatibility complex (MHC) class I associated chains A and B
MyD88: Myeloid differentiation primary response protein 88
NAFLD: Non-alcoholic fatty liver disease
NASH: Non-alcoholic steatohepatitis
NETs: Neutrophil extracellular traps
NKG2D: Nature killer group 2D
NF-κB: Nuclear factor κB
NE: Neutrophil elastase
NKT: Natural killer T cells
NK: Natural killer cells
OPN: Osteopontin
PD-L1: Programmed death ligand 1
PR3: Proteinase 3
PD-1: Programmed death 1
PTEN: Phosphatase and tensin homolog
RFA: Radiofrequency ablation
ROS: Reactive oxygen species
SASP: Senescence-associated secretory phenotype
SCFA: Short-chain fatty acid
STAT: Signal transducer and activator of transcription
TACE: Transarterial chemoembolization
TLR: Toll-like receptor
TGF-β: Transforming growth factor-beta
TNF-α: Tumor necrosis factor-alpha
THR-β: thyroid hormone receptor β
THR-α: thyroid hormone receptor α
TKI: Tyrosine kinase inhibitor
TNFSF14/LIGHT: Tumor necrosis factor superfamily member 14
VEGF: Vascular endothelial growth factor
Virus-HCC: Viral associated hepatocellular Carcinoma
VCAM-1: Vascular cell adhesion molecule-1
VAP-1: Vascular adhesion protein-1
VLDL: Very low-density lipoprotein.
